# Can diet composition estimates using stable isotope analysis of feathers predict growth and condition in nestling mountain bluebirds (*Sialia currucoides*)?

**DOI:** 10.1002/ece3.8210

**Published:** 2021-10-14

**Authors:** Aija F. White, Russell D. Dawson

**Affiliations:** ^1^ Department of Ecosystem Science and Management University of Northern British Columbia Prince George BC Canada

**Keywords:** avian insectivore, diet composition, nestling condition, nutrition, stable isotope mixing models

## Abstract

Insectivorous birds breeding in seasonal environments provision their dependent young during periods when prey diversity and abundance vary. Consequently, the composition and nutritional value of diets parents feed to their offspring may differ within and among broods, potentially affecting the condition of nestlings. In a population of mountain bluebirds (*Sialia currucoides*), we used two methods to estimate diet composition for individual nestlings: direct observation of provisioning using video recordings at 5 and 9 days post‐hatch, and stable isotopes of the δ^13^C and δ^15^N in nestling feathers and prey followed by analysis with mixing models. We determined the macronutrient content (% fat and lean mass) and estimated the metabolized energy from each type of prey. We evaluated whether different methods of estimating diet composition would produce similar results, and whether the types of prey nestlings ate at one or both ages affected their morphology, growth rates, or blood ketone concentration. We found that bluebirds fed their young 5 main types of prey: beetles, cicadas, grasshoppers, insect larvae, and spiders. Both observational and mixing model estimates of diet composition indicated that larvae are traded off with grasshoppers and that fewer larvae are provided to nestlings as the season progresses. In evaluating how diet influences individual growth and condition, estimates from direct observations had greater explanatory power than those from mixing models, indicating that diets rich in the most energy‐dense prey (greatest fat content; cicadas and larvae) were associated with larger size and higher body condition, and faster rate of mass gain and growth of tarsus. Lower value prey had more limited, specific effects on nestlings, but may still be important dietary components. While isotopic methods produced estimates of diet composition that were generally informative, when applied to explain the growth and condition of nestlings they proved less useful.

## Graphical abstract

Insectivorous birds breeding in seasonal environments provision their dependent young during periods when prey diversity and abundance vary, potentially affecting the growth and condition of nestlings. We used two methods to estimate diet composition for individual mountain bluebird (*Sialia currucoides*) nestlings: direct observation of provisioning using video recordings at 5 and 9 days post‐hatch, and stable isotopes of the δ^13^C and δ^15^N in nestling feathers and prey followed by analysis with mixing models. We found that bluebirds fed their young 5 main types of prey (beetles, cicadas, grasshoppers, insect larvae, and spiders) and that estimates from direct observations had greater explanatory power than those from mixing models, indicating that diets rich in the most energy‐dense prey (greatest fat content; cicadas and larvae) were associated with larger size and higher body condition, and faster rate of mass gain and growth of tarsus.

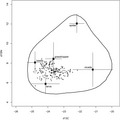

## 1 INTRODUCTION

Generalist avian foragers eat a variety of prey, which may differ in caloric value and micronutrient content. When the types of prey available vary seasonally and/or spatially, inter‐individual differences in diet composition, and thus energetic and micronutrient intake, are likely to result (Guglielmo et al., 2017; Nour et al., 1998; Smith & McWilliams, 2009). Generalist insectivorous birds breeding in temperate regions are especially likely to experience seasonal variation in prey availability and therefore diet composition (Bolduc et al., 2013; Eeva et al., 2000). While short‐term caloric restriction or nutritional deficiency may be relatively benign for adults in reasonable condition, when experienced by juveniles early in development, the consequences may be much more severe (Krause et al., 2009; McCue, 2010).

Many studies of the relationships between diet and the condition of nestlings are conducted on species with relatively narrow dietary niches, or focus on specific types of prey rather than diet composition more broadly (e.g., Arnold et al., 2007, 2010; Burger et al., 2012; McGraw et al., 2002). Such studies have limited application to understanding how diet composition may affect offspring condition among generalist insectivores, and there is less consensus about the relative importance of diet composition to the performance of offspring in such species. Since they are adapted to consume a variety of prey types and should more easily resort to prey switching, it is possible that generalists are less sensitive to shifts in diet composition than specialists are. Given that insectivorous birds continue to be of conservation concern in many ecosystems (Rosenberg et al., 2019), and insect populations are generally thought to be in decline (van Klink et al., 2020; Wagner, 2020; but see Crossley et al., 2020), a clearer understanding of how diet alters nestling condition in such species has clear relevance for management and conservation policy.

The primary difficulty associated with evaluating diet composition is accurately estimating the proportional contributions of each food source to the overall quantity ingested by a consumer. Traditionally, direct sampling of consumption (via observations, fecal samples, gut contents, etc.) has been the approach used to produce diet composition estimates, and these methods continue to be successfully employed by many researchers (Jenni et al., 1990; Tanneberger et al., 2017; Yoshikawa & Osada, 2015). Because generalist foragers have more diverse diets than specialists, more observations may be required to reliably estimate diet composition when using these methods to quantify the diet of a species with a broad dietary niche.

A more recent development to indirectly determine diet composition is the use of stable isotope analysis (SIA; DeNiro & Epstein, 1978; Vogel, 1978). SIA assays food sources and consumer tissues to produce an isotopic “signature” (isotopic ratios of tracer elements), which stable isotope mixing models (SIMM) use to identify the most likely combination of food sources that would result in the signature of the consumer, after adjusting for trophic enrichment in consumers (Phillips & Gregg, 2001; Schwarcz, 1991). There may be significant uncertainty associated with SIMM estimates of diet composition, particularly for generalist foragers that have many potential food sources, and thus a greater variety of potential diet compositions that could produce the isotopic signature of a given consumer (Moore & Semmens, 2008; Parnell et al., 2013; Phillips & Gregg, 2003). The certainty of estimates produced from SIMM may be greatly increased if investigators have some prior knowledge of the proportions of different prey sources in the diets of individuals being assayed (e.g., Derbridge et al., 2015). Used in SIMM as informative priors, these limit the potential solutions that may be possible in theory, but which are unlikely in reality. However, strong informative priors may have a disproportionate influence on diet estimation when isotopic data are uninformative or in conflict with priors (e.g., Robinson et al., 2018), and it is essential to consider whether the technique used to generate priors is itself biased (Swan et al., 2020).

While both direct (observational) and indirect (SIA) methods may be successful, they differ in one key respect: direct methods are often relevant only for brief sampling periods (Storms et al., 2008; Wiebe & Slagsvold, 2014), while stable isotope signatures can represent diets over longer periods of time, depending on the rate of isotopic turnover in tissues used for analysis (Bond et al., 2016; Hobson & Bairlein, 2003). In research on birds, stable isotope analysis of feathers has proven exceptionally useful as a tool to retroactively determine diet composition, because the isotopic signature of feathers is determined at the time they are grown, and does not change until the feather is replaced (Hobson & Clark, 1992a; reviewed in Inger & Bearhop, 2008). Additionally, feathers are generally retained on the body for defined periods of time (according to a seasonal molt cycle), and so among adult birds, feathers represent diet following the most recent episode of molting while feathers were regrown, whereas among juveniles, feathers should be indicative of diet early in life (Pagani‐Núñez et al., 2017; Swan et al., 2020).

In application, studies that use short‐term estimates may identify a critical prey item or nutrient correlated with nestling performance, while SIA may identify more general, integrated patterns in diets. Taken separately, it is difficult to document connections between specific prey items fed at different points in nestling development, and their relevance to success over a longer period of time, in addition to overall diet composition. To better characterize nutritional “pinch points” or key prey items that affect nestling condition and success, while still capturing diet composition over a longer developmental time frame with less uncertainty, an approach that considers both point and summary estimates is warranted. Such comprehensive assessments may reveal how short‐term observations reflect long‐term trends, overarching ecological constraints, and perhaps the predictive or informative utility of focal observations of feeding relative to more general estimation methods.

In a population of breeding mountain bluebirds (*Sialia currucoides*), we recorded provisioning visits made by parents to nestlings, generating point estimates of diet composition for individual nestlings at two different ages. We also measured nestlings during the course of the brood‐rearing period to evaluate their growth and condition, and collected feathers from nestlings late in the brood‐rearing phase for SIA. The isotopic signatures of feathers from nestlings were used in SIMM, informed by the point estimates of diet composition generated by provisioning observations, to quantify the diets of nestlings. We chose to use feathers as the tissue of interest because nestling bluebirds hatch with down feathers (neossoptiles) at a few sites on the body (Johnson & Dawson, 2020), but their body and flight feathers, including those we sampled (teleoptiles from the spinal tract), grow in after hatch, while nestlings are being provisioned by their parents. The pattern of feather growth in mountain bluebirds resembles that of close relative eastern bluebirds (*Sialia sialis*): spinal tract pinfeathers are visible under the skin by three days post‐hatch, but development continues until feathers fully unsheathe by 10 or 11 days after hatch (Pinkowski, 1975). Thus, feathers from the spinal tract are likely to reflect diet between days 2 and 10 following hatch, and we were interested in determining whether SIMM diet estimates derived from feathers may provide unique insights not provided by point estimates generated from relatively brief observations during brood rearing. Our objectives were to: (i) characterize the diets fed to nestlings at different points in their development; (ii) estimate diet integrated over a longer time period, by using SIMM to evaluate isotopic signatures of nestling feathers; (iii) assess the differences in estimates of dietary composition (proportional contributions of each prey group) produced from SIMM, relative to observational estimates; and (iv) identify whether diet composition estimates from either dataset (observational or SIMM) showed relationships between different types of prey and the condition of nestlings.

## 2 METHODS

### 2.1 Site and species description

We studied mountain bluebirds breeding in nest boxes near Williams Lake, British Columbia, Canada (51°N, 122°W; 700 ‒ 1100 m a.s.l.; see O’Brien & Dawson, 2008, for further site details), in 2016. The habitat is primarily open grassland, with scattered forest and riparian areas, dominated by Douglas‐fir (*Pseudotsuga menziesii*) and trembling aspen (*Populus tremuloides*), respectively (Wikeem & Wikeem, 2004). Nest boxes were installed in pairs to reduce interspecific competition for nesting sites (Wiebe, 2016), with ~5 m between boxes in each pair. Mountain bluebirds are territorial during the breeding season, and typically forage within 100 m of their nest box while provisioning offspring (Power, 1980), so each box pair was generally separated ≥200 m from the next pair, and was considered a distinct territory. A total of 86 breeding territories with nest boxes were available and monitored. Mountain bluebirds generally returned to the study site in mid‐March and initiated egg laying beginning in mid‐April (Johnson & Dawson, 2020). Females lay one egg per day and typically begin incubation on the day the penultimate egg is laid (Johnson et al., 2013). Clutches of 5 or 6 eggs are most common, and females incubate them for approximately 13 days before hatching occurs (Johnson & Dawson, 2020). Both parents feed nestlings a variety of arthropods (insects and spiders) during brood rearing, and fledging occurs at 18–22 days post‐hatch. Female bluebirds are facultatively double‐brooded (Johnson & Dawson, 2020), and we sampled nestlings from both first and second broods for this study to better characterize seasonal shifts in diet.

### 2.2 General field procedures

We monitored nest boxes from mid‐April to early August. At the beginning of the season, we checked nest boxes on alternate days, and once a nest was nearly complete, we visited boxes daily to document egg laying and confirm clutch completion. No visits were made to nests during incubation until the earliest predicted hatch date, 12 days after the last egg was laid. On the first day after eggs had hatched (hatch day was considered day 0 of brood rearing), we weighed each nestling on an electronic balance (±0.01 g), and used a non‐toxic marker to uniquely identify each individual. Subsequently, from days 3 to 15, we weighed nestlings with a spring scale (± 0.125 g) and measured their right tarsus using digital calipers (± 0.01 mm) every 2 days. On day 13, for the majority of broods (*n *= 109 nestlings, from 37 broods), we collected ~15 μl of blood via venipuncture of the brachial vein of each nestling to measure blood ketone (β‐hydroxybutyrate, hereafter β‐OH) concentration using a portable monitor (Freestyle Neo, Abbott Pharmaceuticals, Inc.), as an indicator of nutritional stress (Albano et al., 2011). On day 15, we also determined the sex of nestlings by plumage color, and measured the lengths of the combined head and bill with digital calipers (hereafter “head–bill”; ± 0.01 mm) and eighth primary flight feather length with a ruler (± 0.5 mm). At this time, we also collected 4–5 pinfeathers from the spinal tract of each nestling, which were stored in opaque envelopes prior to processing for stable isotope analysis. All protocols involving animals were approved by the University of Northern British Columbia Animal Care and Use Committee (protocol 2016–11).

We calculated growth rate constants using a Gompertz model for tarsus, and a logistic model for mass, following the methods of Dawson and Bidwell (2005). To generate a variable to represent general structural size for each individual, we conducted a principal components analysis on day 15 tarsus, head–bill, and eighth primary feather length, and extracted the first principal component ([PC], which explained 60.6% of the variance) as an index of structural size. Positive values of this PC were indicative of generally larger structural size, as all 3 variables loaded positively (matrix weights: tarsus = 0.56, head–bill = 0.65, eighth primary feather = 0.51). We used the residuals of a regression of day 15 mass on the structural size PC as a measure of size‐adjusted mass (i.e., body condition) for each nestling.

### 2.3 Feather processing for stable isotope analysis

We processed feathers for SIA by soaking them in a 2:1 chloroform:methanol solution for 1 h, decanting the solution, then allowing them to air‐dry for 24 h in a fume hood. Feather barbs were cut away from the rachis, and coarsely homogenized, and 0.35 ± 0.02 mg was weighed into tin capsules (5 × 3.5 mm D2303, Elemental Microanalysis). SIA was conducted on feathers from a total of 190 nestlings in 54 broods, though some individuals were later excluded due to high proportions of uncommon prey in their diets (see below). Additionally, feathers from the smallest nestling from each brood were not collected for SIA due to the potential confounding effects of nutritional stress on stable isotope signatures (Hobson & Clark, 1992b).

### 2.4 Feeding observations

Beginning in April prior to egg laying, we installed “dummy” cameras within the interior of nest boxes. Operational cameras (HawkEye HD, Birdhouse Spy Cams) were placed inside nest boxes to record provisioning by parents on days 5 and/or 9 of brood rearing. Recordings were made at both ages for 49 broods (*n* = 170 nestlings); an additional 4 broods were recorded only once (*n *= 1 brood of 4 nestlings on day 5 only, and 3 broods, totaling 12 nestlings, on day 9 only). Prior to the start of each recording session, we used water‐based paint to uniquely mark the head of each nestling. The first half hour of each recording was discarded to ensure parents had acclimated to any disturbance caused by camera set up and marking of nestlings, and had resumed normal provisioning; all parents who fed regularly returned to feed within this period. We used the following 3.5 h segment of each recording for analysis, as robust regression showed that per capita provisioning rates estimated from 3.5 h recordings were very similar to those from longer recordings (5–7 h; *F*_1,24_ = 290.83, *P *< .01, adjusted *r* ^2^ = 0.92; A. White, *unpubl*. *data*). For each provisioning event, the sex of the parent was determined using plumage color, head markings (see above) were used to identify which of the nestling(s) were fed, and prey item(s) were identified to taxonomic order. The sizes of prey items were scored relative to the area in profile of the beaks of parents following Schwagmeyer et al. (2002), with small items being ≤ half the area of the beak, large items ≥1.5 times the beak area, and medium items being > half but <1.5 times the area of the beak in profile.

### 2.5 Voucher prey specimens

We chose the orders of arthropods to be used as voucher specimens based on provisioning recordings made in previous seasons. We prioritized the 5 most common prey types fed to nestlings: beetles (Coleoptera), cicadas (Hemiptera: Cicadidae), grasshoppers (Orthoptera: Acrididae), insect larvae (mostly Lepidoptera: Noctuidae), and spiders (Araneae). Together, these comprised 89.8% of identifiable prey items (*n *= 5744), and 91.4% of the estimated biomass from 7198 provisioning events in 2015. We chose the taxonomic families of voucher specimens in most cases based on provisioning recordings, but for two groups that were not well resolved on recordings (spiders and beetles), we used the most commonly collected groups in pitfall traps and sweep‐net samples that were also observed on recordings (Lycosidae and Tenebrionidae, respectively). We hand‐collected or sweep‐netted all voucher insects on the field site between May and July 2016, euthanized them immediately after capture with ethyl acetate vapor, and froze them at −20°C within 8 h of collection. They were later thawed and weighed on an analytical balance (± 0.0001 g), and sorted into size classes matching those in provisioning observations (see above). The average mass of each prey type in each size class was used to estimate the biomass delivered and the proportional contribution of each type of prey to the diet of each nestling (see below).

### 2.6 Voucher specimen processing for stable isotope analysis

We selected specimens of each of the 5 common types of prey to process for SIA (*n *= 5–10 per type; total = 33). The remainder of the specimens were used to determine the macronutrient content and estimate the caloric value of prey commonly fed to nestlings (see Appendix 1). We processed prey items (of the 5 types listed above) for SIA by removing the digestive tract from each specimen to prevent interference from gut contents (largely plant material, as most of the prey are herbivorous). While this is not common practice, our rationale was that it is unlikely that plant materials can be digested and absorbed by nestling bluebirds: birds lack the ability to endogenously produce cellulase (Karasov & Douglas, 2013), and insectivores generally show very low digestive efficiency for plant tissues (Castro et al., 1989). After removing the digestive tracts and contents from each prey item, we rinsed the remaining tissues with distilled water. We then halved each sample lengthwise: one half was used for δ^15^N determination, and required no processing other than freeze‐drying and homogenizing prior to encapsulating (see below); the other half was lipid‐extracted prior to freeze‐drying. Lipid extractions were carried out to determine δ^13^C more accurately, as lipid content may bias δ^13^C measurement in invertebrate tissues, and may alter the outcomes of SIMM when sources differ substantially in lipid content (Post et al., 2007). We performed lipid extraction by coarsely homogenizing the designated samples and soaking them in a 2:1 chloroform:methanol solution for 2 h, in stoppered glass vials (Perkins et al., 2013). The solution was then decanted and replaced with fresh solution, followed by another 2‐h soaking; this solution was again discarded, and samples were left to air‐dry in a fume hood for 24 h. The lipid‐extracted samples and their matched non‐extracted equivalents were subsequently freeze‐dried for 24 h, and homogenized to a fine powder using an agate mortar and pestle. Tools were rinsed with ethanol and distilled water between each sample. We weighed 0.35 ± 0.02 mg of each homogenized sample and placed it in a tin capsule (5 × 3.5 mm D2303, Elemental Microanalysis).

### 2.7 Stable isotope analysis

The isotopic ratios and concentration of C and N (δ^13^C, δ^15^N, [C], and [N]) in feather and prey samples were determined with a continuous flow Costech 4010 EA‐Delta V Plus isotope ratio mass spectrometer at the Laboratory for Stable Isotope Science at Western University, Ontario, Canada. International δ^13^C and δ^15^N standards USGS40 and USGS41a were included at a ratio of 1:10 samples. Internal standards (2 × IAEA‐CH‐6 and 5 x keratin powder) were included in each analytical session (40 samples/session) to monitor instrument drift. Isotopic ratios of carbon and nitrogen are expressed as δ values (‰) in parts per thousand, relative to Vienna PeeDee Belemnite (VPDB), or atmospheric N_2_ (AIR) standards as follows: δX = (ratio of sample/ratio of standard) − 1. The coefficient of variation among analytical sessions was 0.1% for δ^13^C and 0.6% for δ^15^N.

### 2.8 Diet composition estimates

We estimated the proportional contribution of 6 prey types (5 common types and “other,” which were uncommon or unidentified prey) to the overall amount of biomass consumed by each nestling in provisioning recordings on days 5 and 9 of the brood‐rearing period. We multiplied the number of items of each of the 5 common prey types fed to each nestling at each age by the average mass of voucher specimens of the same size (see above). To determine the proportion of biomass contributed by unknown or uncommon items, we used the average mass of all common prey items of the same size class. The estimated biomass of the 5 main prey types, and any uncommon and unidentified prey, was summed to estimate the total biomass consumed by each nestling, and the proportional contribution of each type of prey was determined by dividing the mass of each type by the total estimated biomass consumed by a given nestling. Nestling identity was known in 98.7% of feeding events, and for the few events where unknown nestlings were fed, we divided the biomass of the item equally among all nestlings in the brood.

For the 186 nestlings in provisioning recordings made on day 5 and/or 9, in 74.2% of observations (264 of 356 observation records of individual nestlings), the contribution of unidentified and uncommon prey biomass to the estimated total consumed by each nestling was ≤5%. For these records, we removed unidentified and uncommon items from the total biomass consumed before estimating the proportional contributions for the 5 common types. For the 92 records of nestlings with >5% of total estimated biomass consumed from unidentified prey items (mean ± standard error [SE]* *= 7.5 ± 1.3% of total estimated biomass; range: 5.2%‒30.3%), we used the method of Robinson et al. (2015) to allocate unknown items to one of 6 prey types (5 common and “other,” uncommon prey) before estimating dietary proportions. Briefly, this method simulates the most probable allocation of unidentified items among all possible prey types, based on the proportions of identified prey of each type delivered to an individual during the recording. The best solution identified during the simulations was then used in calculating the proportions of biomass of each prey type using voucher sample masses, as detailed above. We then calculated the dietary proportions of the 5 main prey types for nestlings that consumed <5% of estimated biomass from uncommon prey after removing the biomass contributed by the “other” category, as detailed above. Individuals that had >5% of estimated biomass consumed from the “other” category at this point were excluded from further analyses, leaving a sample of 162 nestlings from 50 broods whose diets were eligible for estimation using SIMM.

### 2.9 Stable isotope mixing models

The 5 common prey types used as sources in mixing models represented 95.4% of items observed in the 4102 feeding events where prey were identified in provisioning records. For the nestlings selected for diet estimation using SIMM, the 5 common prey types accounted for ≥95% of the estimated biomass consumed. While it was clear that the 5 sources we selected would be appropriate to model the diets of the majority of bluebird nestlings on our study site, we also evaluated the suitability of our sources for SIMM by determining whether they would need aggregating *a priori*, due to significant overlap in their isotopic composition (Phillips et al., 2013). For the source isotopic signatures, we used the δ^13^C and [C] values from lipid‐extracted samples, and δ^15^N and [N] from non‐extracted samples, and conducted a discriminant function analysis with leave‐one‐out classification to determine whether prey types could be differentiated using δ^13^C, δ^15^N, [C], and [N]. All cicadas, grasshoppers, and spiders were always assigned to the correct group; one beetle and one larva were both miscategorized as grasshoppers, for an overall error rate of 6.08%. Post hoc multiple analysis of variance confirmed that isotopic characteristics differed among prey groups (Pillai's trace = 2.14, *F*_16, 144_ = 10.40, *P *< .01, residual *df* = 36). As a consequence, we did not aggregate any of our sources prior to using them in SIMM.

Mixing models for diet estimates were constructed using the MixSIAR package (Stock et al., 2018) in R (R Core Development Team 2018), and we selected trophic enrichment factors of Δ^13^C = 2.7 ± 0.1 and Δ^15^N = 4.0 ± 0.1 ( $\bar{x}$ ± *SD*; Hobson & Bairlein, 2003) after comparing the performance of values from the literature, using the methods of Smith et al. 2013 (see Appendix 1). MixSIAR models use a Bayesian framework and Markov Chain Monte Carlo simulations to generate estimates of the proportional contribution of sources to the diet of consumers. We used the “long” setting (3 chains, 300,000 iterations each, burn‐in of 200,000 iterations) for concentration‐dependent models.

In observations, diet composition had low repeatability between ages and differed substantially even among individuals within broods during a given observation session (calculated as per Lessells & Boag, 1987; *r* ranged from 0.12 to 0.53 for different age groups and prey types). Due to this substantial inter‐individual and among‐brood variability, we used brood identity and individual identity (nested in brood) as random effects. The mixing models were configured to allow for residual error, which permits variation in the physiological integration of isotopes into consumer tissues, and process error, which allows for variation in the isotopic signatures of sources.

We used MixSIAR to generate diet composition estimates twice, first with a uniform prior (the default in MixSIAR, where α = 1 for all 5 prey types) and one with an informative prior derived from provisioning observations. The feathers we sampled are grown between days 2 and 10 of brood rearing, and provisioning observations were carried out during this period, on days 5 and 9. To generate the informative prior, we averaged between day 5 and day 9 observations the proportion of biomass contributed by each of the 5 common prey types across all broods, and rescaled this to match the uninformative prior (thus, α = 0.35, 1.1, 1.55, 1.6, and 0.4 for beetles, cicadas, grasshoppers, larvae, and spiders, respectively). We compared the leave‐one‐out cross‐validation information criterion values (LOOic) between the uninformed and informed SIMM, and elected to use the estimates provided by the informed SIMM for further analyses, as the LOOic value of the informed model was lower (uniform prior SIMM LOOic = −244.8, informed prior SIMM LOOic = −246.3). From the informed SIMM, we extracted the estimated proportion of each prey type in the diet of each consumer (nestling).

### 2.10 Statistical analyses

Because of the collinear nature of proportion data (i.e., prey types always sum to 1), we log‐ratio‐transformed all diet composition variables used as predictors (Aitchison, 1999). We first evaluated whether feather δ^13^C and δ^15^N showed a relationship with diet estimates from provisioning observations, using generalized linear mixed‐effect models (GLMMs). We generated two models for each element, using as predictors the proportion of biomass from each of the 5 main prey types parents provided, from either day 5 or day 9 provisioning observations. Nestling sex was used as an additional fixed effect, since there may be metabolic differences between female and male nestlings (Johnsen et al., 2003; Love et al., 2005). We used a normal distribution with an identity link for δ^13^C and a log link for δ^15^N. A log link was used for δ^15^N because the residuals from an identity link model were clustered, and this was resolved by the use of a log link function. We then evaluated whether SIMM‐estimated proportions of each prey type were different from observations made on day 5 and day 9, using Wilcoxon signed‐rank tests.

To evaluate general trends in diets, we used principal components analysis (PCA) to reduce the dimensionality of the proportions of prey types in the diets of nestlings. We conducted two PCAs, one with observation‐derived estimates of diet (from both day 5 and day 9) and one with the estimated proportions of prey generated by SIMM. We retained the first component (PC1) from each analysis, according to the broken‐stick model (Frontier, 1976; we use “PC1_OBS_” to denote the PC1 generated from observational diet estimates, and “PC1_SIMM_” to indicate the PC1 generated from SIMM estimates). We evaluated age and seasonal effects on diet with linear mixed‐effect models (LMM), using either PC1_OBS_ or PC1_SIMM_ as a response variable. The date of each observation (where 1 = January 1), nestling age, a date*age interaction, and nestling sex were used as fixed effects in models of PC1 _OBS_, but because there was no observation date *per se* for SIMM estimates, we used the hatching date of the brood and the sex of each individual, but could not include age as a predictor in modeling PC1_SIMM_. Brood identity and nestling identity were used as random effects. These models used restricted maximum‐likelihood estimation, and calculated degrees of freedom using the method of Kenward and Roger (1997). Model fits were assessed with observed‐versus‐fitted and residual plots; significance of these models was determined with *F* tests.

We then assessed how diet may affect nestling size and condition, using estimates of the proportions of prey in nestling diets as predictors of structural size (PC1 from the PCA of day 15 lengths of tarsus, head–bill, and eighth primary flight feather; see above), size‐adjusted day 15 mass, mass and tarsus growth rate constants, and day 13 blood ketone concentration of nestlings (we did not sample all nestlings for ketones, so sample sizes differ among analyses). We constructed three GLMMs for each of these variables, using as predictors the proportion of each prey group in the diet of each nestling (log‐ratio‐transformed), generated from provisioning recordings at two ages (hereafter OBS_5_ and OBS_9_) or from the mixing model (SIMM). We chose distributions and link functions for response variables as follows: Gaussian distribution and log link for structural size PC1 (generated from day 15 head–bill, tarsus, and eighth primary feather length) and size‐adjusted mass, and Poisson distribution and log link for mass growth rate, tarsus growth rate, and blood ketone concentration. Brood identity was used as a random effect in all models. We used robust variance estimation for fixed effects and an unstructured covariance matrix, and model fit was assessed with observed‐versus‐fitted and residual plots. Significance of GLMM was determined with Wald tests.

We used Stata 14 (StataCorp 2015) for all analyses other than diet estimation using SIMM.

## 3 RESULTS

**FIGURE 1:**
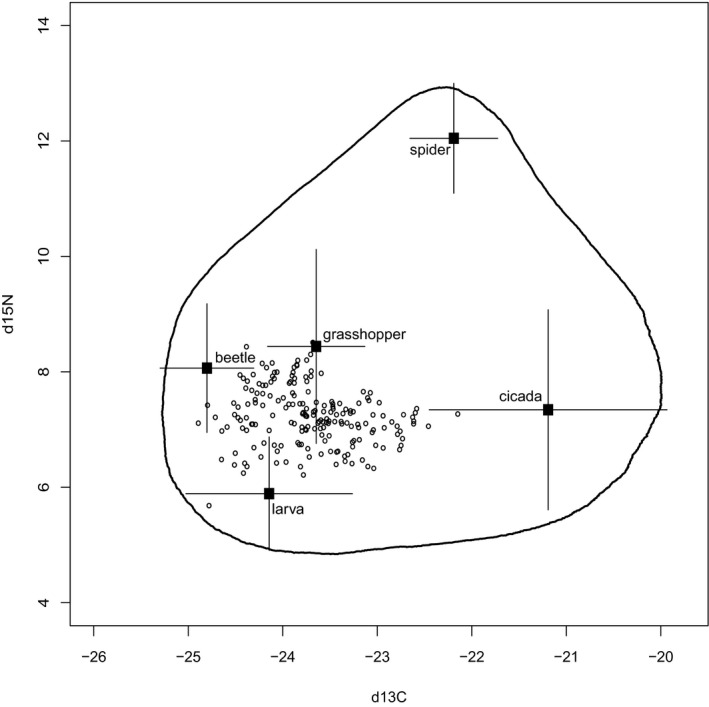
Nestling mountain bluebird (consumer) isotopic signatures (open circles), prey sources (mean ± SD; black squares), and simulated 95% mixing region (contour line), using trophic enrichment factors (Δ^13^C = 2.7 ± 0.1 and Δ^15^N = 4.0 ± 0.1) from Hobson and Bairlein (2003)

The five common prey types observed collectively comprised 95.4% of items provided in all provisioning recordings: grasshoppers (35.5%), insect larvae (25.9%), beetles (15.9%), cicadas (13.1%), and spiders (5%). In terms of biomass, grasshoppers, larvae, and cicadas were the most substantial contributors (collectively 84% of all estimated biomass delivered). Isotopically, most of these sources were well resolved and distinct from each other, with the exception of grasshoppers and beetles (Table 1; Figure 1). The isotopic signatures of food sources were consistent with the dietary patterns of each group: spiders, the most predatory prey type, were the most enriched in δ^15^N, while insect larvae (caterpillars) and cicadas, the most herbivorous, were the least enriched. The more facultative omnivores, beetles and grasshoppers, had intermediate values of δ^15^N.

**TABLE 1:** Isotopic characteristics (mean ± standard deviation [SD]) of prey types commonly consumed by nestling mountain bluebirds (*Sialia currucoides*). Specimens were collected near Williams Lake, BC, Canada, in 2016

Source	$\bar{x}$ δ^13^ *C*	*SD* δ^13^C	$\bar{x}$ δ^15^N	*SD* δ^15^N	$\bar{x}$[C]	$\bar{x}$[N]	*n*
Beetle	−27.50	0.40	4.06	1.02	0.48	0.10	5
Cicada	−23.89	1.16	3.34	1.63	0.46	0.11	10
Grasshopper	−26.35	0.42	4.44	1.58	0.44	0.11	8
Lepidoptera larvae	−26.84	0.78	1.89	0.88	0.42	0.10	5
Spider	−24.89	0.36	8.05	0.85	0.44	0.13	5

Nestling feather δ^13^C was negatively related to the proportion of grasshoppers consumed early in brood rearing (day 5 diet: Wald $\chi_{6}^{2}$ = 14.85, *P* = .02; grasshoppers: *z* = −2.57, *P* = .01; all other prey, *P* > .16). Additionally, greater ^13^C enrichment was associated with consuming more cicadas on day 9 (day 9 diet: Wald $\chi_{6}^{2}$ = 19.73, *P* = .003; cicadas: *z* = 2.11, *P* = .01; all other prey, *P* > .16). Male nestlings were more enriched in δ^13^C than females in both models (day 5: sex *β* = −0.09 ± 0.04; day 9: sex *β* = −0.07 ± 0.03; both models: *z* = −2.22, *P* = .03). Feather δ^15^N did not differ between the sexes, but nestlings that consumed more beetles on day 5 were more enriched in ^15^N (day 5 diet: Wald $\chi_{6}^{2}$ = 19.55, *P* = .003; beetles: *β* = 0.03 ± 0.008, *z* = 3.50, *P *< .001). Feather δ^15^N was also lower among nestlings observed consuming more cicadas in day 5 observations (*β* = −0.01 ± 0.006, *z* = −2.08, *P* =.04; all other predictors, *P* > .1). Greater consumption of spiders on day 9 was also associated with decreased feather δ^15^N (day 9 diet: Wald $\chi_{6}^{2}$ = 12.70, *P* = .048; spiders: *β* = −0.008 ± 0.003, *z* = −2.71, *P* = .007; all other predictors, *P* > .12).

SIMM estimates differed from observational estimates in some, but not all, cases (Table 2). For cicadas and larvae, SIMM estimates did not differ from the proportions observed in recordings of nestlings at either age (cicadas: OBS_5_ vs. SIMM: *z *= −0.421, *P* = .67, OBS_9_ vs. SIMM: *z *= 0.96, *P* = .34; larvae: OBS_5_ vs. SIMM: *z *= −0.579, *P* = .56; OBS_9_ vs. SIMM: *z *= −1.10, *P* = .27). SIMM estimates of grasshopper consumption did not differ from observations made on day 5, which were significantly lower than estimates generated from day 9 observations (OBS_5_ vs. SIMM: *z *= −1.21, *P* = .23, OBS_9_ vs. SIMM: *z *= −4.707, *P* < .001). SIMM revised upward the estimated contribution of beetles to nestling diets (OBS_5_ vs. SIMM: *z *= 8.58, *P* < .001; OBS_9_ vs. SIMM: *z *= 9.93, *P* < .001) and substantially decreased the proportion of spiders in diets relative to what was observed in recordings at both ages (OBS_5_ vs. SIMM: *z *= −8.32, *P* < .001; OBS_9_ vs. SIMM: *z *= −5.86, *P* < .001).

**TABLE 2:** Mean ± SD of the proportions of the 5 common prey types consumed by nestling mountain bluebirds (*Sialia currucoides*) near Williams Lake, BC, Canada, in 2016, estimated directly from observations on days 5 and 9 of brood rearing (day 0 = hatch day; *n *= 145 nestlings from 44 broods on day 5, 167 nestlings from 50 broods on day 9) and from a stable isotope mixing model (SIMM; *n *= 162 nestlings from 50 broods)

	Prey type ($\bar{x}$ ± *SD* %)				
Source of estimate	Beetles	Cicadas	Grasshoppers	Larvae	Spiders
Observations, day 5	7.9 ± 14.5	21.6 ± 26.9	26.1 ± 26.6	31.5 ± 27.4	12.9 ± 14.4
Observations, day 9	5.1 ± 9.5	22.6 ± 29.4	35.3 ± 30.1	32.8 ± 27.3	4.0 ± 5.5
SIMM	25.1 ± 12.5	24.1 ± 16.4	20.6 ± 12.1	29.5 ± 16.7	0.7 ± 0.2

The PCs describing diet produced from observed (PC1_OBS_) and SIMM estimates (PC1_SIMM_) showed different relationships among the most common prey types, grasshoppers and larvae, and other types of prey (Table 3). Where PC1_OBS_ showed a negative loading for grasshoppers and a positive loading for larvae, the opposite was true for PC1 _SIMM_. As a consequence, positive values of PC1_OBS_ indicated diets with more larvae and fewer grasshoppers, while positive values of PC1_SIMM_ described diets with fewer larvae and more grasshoppers. In both PCs, beetles and spiders loaded positively and cicadas loaded negatively, meaning that for both PCs, positive values were also indicative of diets high in beetles and spiders, and low in cicadas.

**TABLE 3:** Loadings for diet composition principal components produced from observed (PC1_OBS_) and SIMM (PC1_SIMM_) estimates of the proportional contributions of 5 common prey types to the diets of nestling mountain bluebirds (*Sialia currucoides*), near Williams Lake, BC, Canada, in 2016

Prey type (proportion of total biomass)	PC1_OBS_	PC1_SIMM_
Beetles	0.373	0.484
Cicadas	−0.534	−0.581
Grasshoppers	−0.275	0.508
Larvae	0.625	−0.405
Spiders	0.332	0.086
Variance explained (%)	34.2	53.8

When these PCs were used as response variables to assess the effects of date, nestling age, and sex on diet composition, both models revealed similar changes in larvae and grasshoppers, but not other types of prey, over time (PC1_OBS_ model *F*_4, 220.11_ = 13.53, *P *< .001; PC1_SIMM_ model *F*_2, 147.08_ = 3.58, *P* = .03). Date had a negative effect on PC1_OBS_, indicating that nestlings consumed fewer larvae, spiders, and beetles, and more grasshoppers and cicadas, over the course of the season (date: *β* = −0.024 ± 0.01, *t* = −2.27, *P* = .024). PC1_SIMM_ was positively affected by date, which also indicated that diets became poorer in larvae and richer in grasshoppers over the course of the season (date: *β* = 0.003 ± 0.001, *t* = 2.52, *P* < .001). However, due to differences in the matrix loadings for cicadas, beetles, and spiders (Table 3), the model for PC1_SIMM_ suggested that nestlings consumed more beetles and spiders, and fewer cicadas, as the season progressed. Additionally, PC1_OBS_ decreased with nestling age (age: *β* = −0.23 ± 0.10, *t* = −2.21, *P* = .03), and neither model showed an effect of sex on diet (both *P* > .31).

All of the metrics of nestling growth and condition evaluated showed links to diet composition, but the types of prey and their effects on nestlings varied depending on the estimates used as predictors (see Table 4 for model estimates for all significant effects). Nestling structural size was positively associated with the consumption of all prey other than spiders, which were negatively associated with structural size, when SIMM diet estimates were used as predictors (Wald $\chi_{5}^{2}$ = 43.25, *P *< .001; cicadas, grasshoppers, larvae, and spiders, *P* < .023; beetles, *P* = .05). The model that used day 9 observations as predictors also identified high spider consumption as being associated with reduced structural size, but did not show an effect of any other types of prey (OBS_9_ Wald $\chi_{5}^{2}$ = 424.15, *P *< .001; spiders: *z *= −15.02, *P *< .001; all other prey types, *P* > .4). The model that used OBS_5_ estimates associated increased structural size with higher consumption of beetles and cicadas (OBS_5_ Wald $\chi_{5}^{2}$ = 15.02, *P* = .01; beetles: *z* = 2.20, *P* = .03; cicadas: *z* = 2.97, *P* = .003; all other prey, *P* > .09).

**TABLE 4:** Coefficients ± SE from models evaluating the effect of diet components (5 prey types: beetles, cicadas, grasshoppers, larvae, and spiders) on the characteristics of nestling mountain bluebirds (*Sialia currucoides*), near Williams Lake, BC, Canada, in 2016. Estimates of the proportion of biomass contributed by each prey type were determined via observations on days 5 and 9 post‐hatch (OBS_5_ and OBS_9_) or with stable isotope mixing models (SIMM). Estimates shown are for all predictors that were significant (*P *< .05); “n.s.” indicates no significant effect of a type of prey on a given characteristic

	Nestling characteristics				
	Structural size	Size‐adjusted mass	Mass growth rate	Tarsus growth rate	Blood ketone concentration
Beetles (OBS_5_)	0.82 ± 0.37	9.15 ± 2.93	n.s.	n.s.	n.s.
Beetles (OBS_9_)	n.s.	n.s.	−0.07 ± 0.03	−0.05 ± 0.02	−0.22 ± 0.05
Beetles (SIMM)	34.32 ± 17.73	n.s.	−0.40 ± 0.16	n.s.	−1.36 ± 0.60
Cicadas (OBS_5_)	3.76 ± 1.26	n.s.	0.05 ± 0.02	0.06 ± 0.02	n.s.
Cicadas (OBS_9_)	n.s.	31.69 ± 8.94	0.04 ± 0.02	0.05 ± 0.02	−0.09 ± 0.05
Cicadas (SIMM)	30.60 ± 12.46	66.37 ± 30.08	−0.38 ± 0.18	n.s.	n.s.
Grasshoppers (OBS_5_)	n.s.	n.s.	0.05 ± 0.03	0.06 ± 0.03	−0.10 ± 0.05
Grasshoppers (OBS_9_)	n.s.	0.84 ± 0.37	n.s.	n.s.	−0.21 ± 0.05
Grasshoppers (SIMM)	107.10 ± 45.35	125.98 ± 52.19	n.s.	n.s.	n.s.
Larvae (OBS_5_)	n.s.	n.s.	0.06 ± 0.03	0.08 ± 0.04	n.s.
Larvae (OBS_9_)	n.s.	19.09 ± 6.20	n.s.	0.06 ± 0.03	n.s.
Larvae (SIMM)	95.26 ± 41.23	106.53 ± 41.44	n.s.	n.s.	n.s.
Spiders (OBS_5_)	n.s.	n.s.	0.05 ± 0.03	n.s.	0.14 ± 0.06
Spiders (OBS_9_)	−16.69 ± 1.21	1.74 ± 0.36	n.s.	−0.05 ± 0.02	n.s.
Spiders (SIMM)	−185.58 ± 80.83	n.s.	n.s.	n.s.	n.s.

Diet composition was related to size‐adjusted day 15 mass in all models, but the types of prey identified as important varied. When day 5 diet was evaluated, the proportion of beetles consumed by nestlings had a positive relationship with their size‐adjusted mass, while all other prey types had no significant effect (OBS_5_ Wald $\chi_{5}^{2}$ = 178.42, *P *< .001, beetles: *z* = 3.14, *P* = .002; all other prey, *P* > .7). In contrast, OBS_9_ and SIMM estimates largely agreed in showing that cicadas, grasshoppers, and larvae were all positively associated with size‐adjusted mass at day 15, and beetles were not (OBS_9_ Wald $\chi_{5}^{2}$ = 28.53, *P *< .001, beetles: *P* = .63; all other prey, *P *< .021; SIMM Wald $\chi_{5}^{2}$ = 23.35, *P *< .001; cicadas, grasshoppers, and larvae *P *< .03; other prey, *P* > .29). However, the model that used day 9 observations also identified spiders as being positively associated with body mass, while the model that used SIMM estimates did not (OBS_9_ spiders: *z* = 4.91, *p* < .001; SIMM spiders, *P* > .5). Additionally, the coefficient values in these models showed that while in both cases cicadas and larvae had large effects on day 15 mass, the effect of grasshoppers on day 15 mass differed depending on the estimates used as predictors (Table 4).

Mass growth rates were generally poorly predicted by estimates of diet composition generated from SIMM: No distribution or link function was able to fit these data acceptably (SIMM Wald χ^2^_5_ = 8.92, *P* = .11). In contrast, models of mass growth rates using observational diet composition estimates as predictors fit well (OBS_5_ Wald $\chi_{5}^{2}$ = 14.54, *P* = .01; OBS_9_ Wald $\chi_{5}^{2}$ = 10.99, *P* = .05). In evaluating day 5 diet composition estimates, observations showed that beetles were unimportant (*P* = .92), but mass gain was faster among nestlings that consumed a greater proportion of all other prey (OBS_5_ cicadas: *z* = 2.19, *P* = .029; grasshoppers: *z* = 2.01, *P* = .044; larvae: *z* = 2.26, *P* = .024; spiders: *z* = 1.97, *P* = .049). Observations on day 9 indicated that nestlings consuming more beetles gained mass more slowly; other than a marginal positive influence of cicadas, no other prey types in the diets of nestlings on day 9 showed a significant effect on mass growth rates (OBS_9_ beetles: *z *= −2.41, *P* = .016; cicadas: *z* = 1.86, *P* = .063; all other prey, *P* > .50). Though model fit was generally poor, the SIMM model indicated a negative effect of both beetles and cicadas on mass gain (beetles: *z *= −2.49, *P* = .013; cicadas: *z *= −2.17, *P* = .03).

There was support for an effect of diet on tarsus growth rates when observational estimates from both days 5 and 9 were evaluated (OBS_5_ Wald $\chi_{5}^{2}$ = 16.51, *P* = .006; OBS_9_ Wald $\chi_{5}^{2}$ = 19.85, *P *< .001), but analyses of SIMM estimates provided unreliable information due to generally poor model fit (SIMM Wald $\chi_{5}^{2}$ = 4.58, *P* = .47). None of the types of prey used as predictors predicted tarsus growth rates when SIMM estimates of consumption were modeled (all prey types, *P* > .17). Tarsus growth rates were higher among nestlings fed a greater proportion of cicadas and larvae during observations at both ages (OBS_5_ cicadas: *z* = 3.15, *P* = .002, larvae: *z* = 3.20, *P* = .021; OBS_9_ cicadas: *z* = 2.88, *P* = .004, larvae: *z* = 2.09, *P* = .036). Additionally, beetles and spiders in the diets of day 9 nestlings were negatively associated with tarsus growth rate, but did not influence tarsus growth rates when day 5 diets were evaluated (OBS_5_ beetles and spiders, *P* > .34; OBS_9_ beetles: *z *= −2.17, *P* = .03, spiders *z *= −2.07, *P* = .039). Additionally, the quantity of grasshoppers consumed by day 5 nestlings positively influenced tarsus growth (OBS_5_ grasshoppers: *z* = 2.25, *P* = .025; OBS_9_ grasshoppers, *P* > .48).

Blood ketone concentration (β‐OH; measured in day 13 nestlings), an indicator of nutritional stress, was negatively affected by beetles, but no other types of prey when evaluating SIMM estimates (SIMM Wald $\chi_{5}^{2}$ = 10.95, *P* = .05; beetles: *z* = −2.54, *P* = .01; all other prey types, *P* > .12). Beetles were also associated with lower ketones in the OBS_9_ model, but were not the only significant predictor (OBS_9_ Wald $\chi_{5}^{2}$ = 40.62, *P *< .00; beetles: *z* = −4.44, *P *< .001). Models of day 5 and day 9 observations both showed that increased consumption of grasshoppers was associated with lower blood ketones (OBS_5_ Wald $\chi_{5}^{2}$ = 11.76, *P* = .038; grasshoppers: *z* = −2.04, *P* = .04; OBS_9_ grasshoppers: *z* = −4.26, *P *< .001). The consumption of cicadas among day 9, but not day 5, nestlings was associated with lower ketones, while a greater proportion of spiders in the diets of day 5 nestlings, but not day 9 nestlings, was associated with higher β‐OH (OBS_5_ spiders: *z* = 2.41, *P* = .016; all other prey, *P* > .15; OBS_9_ cicadas: *z* = −1.94, *P* = .05; spiders, *P* > .9).

In assessing the macronutrient profiles of common prey types consumed by bluebird nestlings, we found that while lean tissue as a proportion of total wet mass was relatively similar among all types (25%–31%), grasshoppers had substantially less fat than any of the other types of prey (grasshoppers = 19%; other prey types = 27%–35%; Appendix, Table A1). Cicadas, the item with the greatest average mass, had the highest gross energy content, >30% more energy (kJ) per item than any other prey. However, the second‐ranked prey were Lepidoptera larvae, and due to their higher fat content, they yielded more energy than grasshoppers, despite their lower average mass per item. Beetles and spiders, the smallest prey items, had the lowest energy yields. After adjusting for the metabolic losses in digestion and assimilation (see Appendix 1; Gibb, 1957; Levey & Karasov, 1989), the relative rankings of prey did not vary, but the difference in energy yield between cicadas and insect larvae was reduced to <20%, due to the differences in metabolizable energy coefficients of arthropods with hard exoskeletons that are high in chitin and those with softer, less sclerotized cuticles.

## 4 DISCUSSION

Mountain bluebirds showed substantial variation in diet composition both within and among individual nestlings. While SIMM estimates of diet composition may be accurate and effective for evaluating relationships between nutrition and physical condition in species with less generalist dietary habits, in this study provisioning observations provided more insight regarding how diet composition may influence the physical condition of nestlings. The analyses that used SIMM estimates provided little new information, indicating that the estimates we developed from observations were sufficient to evaluate how diet influences the growth and condition of nestling bluebirds, and to characterize how diets vary among broods and over the course of the breeding season. Both observational and SIMM estimates agreed that insect larvae and grasshoppers are generally traded off in provisioning nestlings and that larvae become less common in diets over the course of the season. However, observational and SIMM estimates provided differing information in regard to the other common prey types (beetles, spiders, and cicadas): Observations showed that more cicadas and fewer beetles and spiders were fed to nestlings as the season progressed, while SIMM estimates indicated the opposite.

In evaluating the relationship between the composition of diets and nestling condition, we found that at one or both ages prey items that are higher in fat and yield the most energy (cicadas and to a lesser extent insect larvae) were associated with better outcomes in all of the metrics of nestling size and growth we evaluated. This result is consistent with optimal foraging theory, which predicts that parents prefer prey with the highest net energy return while provisioning dependent young (Emlen, 1966; Krebs & Cowie, 1976). Additionally, the finding that insect larvae were less abundant in the diets of nestlings later in the season may also be an indicator that searching for these types of prey may become more costly due to declines in their availability, as has been found elsewhere (Burger et al., 2012; Naef‐Daenzer, 2000). While the positive effects of insect larvae on nestling condition have been frequently demonstrated in many similar species (Mägi et al., 2009; Tremblay et al., 2003; Wilkin et al., 2009), the nutritional benefits of cicadas are less well established in the literature, despite being preyed upon by many bird species (Pons, 2020). Grasshoppers, the most common prey type fed to nestlings, showed less substantial links to nestling growth and condition: they were weakly associated with size‐adjusted mass when consumed by day 9 nestlings, and linked to mass and tarsus growth rate only when fed to day 5 nestlings. Given that grasshoppers yield less energy than cicadas and insect larvae, and are only remarkable for their high phosphorus content (Razeng & Watson, 2015) and large body masses they attain late in the season (therefore increased per‐item energetic yield), this is unsurprising.

The prey types with the lowest estimated energetic value, spiders and beetles, were associated with size and growth, but the direction of these relationships depended on the developmental stage of the nestlings consuming them. For example, nestlings fed more spiders early in brood rearing gained weight more quickly, while those fed more spiders on day 9 of brood rearing were of smaller structural size on day 15. Similarly, beetles were linked to larger structural size when consumed in large quantities early in brood rearing, but slower mass gain when they formed a large proportion of the diets of nestlings later on. While both beetles and spiders yield less energy, the caloric needs of younger nestlings are also lower, and these prey types may provide essential amino acids and micronutrients, particularly those needed for nervous system development and bone growth (taurine, K, Ca, and Mg in particular; Arnold et al., 2007; Razeng & Watson, 2015). We suggest that the association of smaller prey types with reduced nestling condition later in the brood‐rearing period is likely the consequence of nestling size, rather than a cause: spiders and beetles are relatively small in size, and thus smaller nestlings are capable of consuming them. For individuals that have larger nest‐mates, these types of prey may be the only ones they can physically accommodate, given that insect larvae (a soft, flexible type of prey) decrease in abundance as the season progresses, and parents transition to feeding older broods with grasshoppers and cicadas, which are hard, inflexible, and larger in size.

Unexpectedly, higher value prey (cicadas and larvae) were not globally associated with decreased nutritional stress (lower blood ketone concentration); reduced ketones were instead linked to greater quantities of beetles, cicadas, and grasshoppers in the diets of nestlings on day 5 and/or day 9. We suggest this may be due to the unusually inclement weather experienced early in the breeding season during the year this study was conducted (A. White, *unpubl. data*). This may have limited the overall quantity of food parents were able to supply, causing a general energetic deficit and reduced condition among all nestlings. Therefore, in broods that were more food‐stressed, even lower quality prey may have been disproportionately beneficial in reducing nutritional stress under atypically adverse conditions.

Our results show that even relatively short‐term observations can produce useful information about diet composition. The analyses that used diet composition estimates from observational data produced results that were logically consistent, and provided insights regarding developmental and seasonal shifts in prey consumption, as well as the importance of different prey types to specific aspects of nestling growth and condition. Provisioning recordings generated data that were sufficient to summarize the major trends in diet, most notably the age‐related and seasonal declines in insect larvae, spiders, and beetles, which are traded‐off with increasing consumption of grasshoppers and cicadas. While SIMM estimates also showed similar seasonal changes in insect larvae and grasshoppers, they predicted an increase in small, low‐value prey (spiders and beetles) and a decrease in large, high‐value prey (cicadas), which conflicts with the typical emergence phenology of cicadas (Jarošík et al., 2011). SIMM estimates also identified a negative effect of cicadas on how quickly nestlings gained mass, which is unlikely given the higher energetic value of cicadas relative to all other prey types (Table A1 in Appendix 1).

SIMM estimates did identify one relationship that was not evident when observational estimates were evaluated, namely a positive effect of grasshoppers and larvae on structural size. Interestingly, the SIMM estimates for grasshoppers and larvae did not differ significantly from the observational estimates, indicating these results may be spurious, the product of inaccurate estimation of the proportional contribution of the other 3 prey types to the diets of nestlings produced by SIMM. Otherwise, the results of analyses that used SIMM largely mirrored those that used provisioning observations, but there were several effects of diet components on size‐adjusted mass, mass and tarsus growth rates, and blood ketone concentration that were not evident when SIMM estimates were used as predictors. Particularly noteworthy was the general lack of model fit observed when SIMM estimates were used to predict the growth rates of body mass and tarsus, which suggests that for species with varied diets, SIMM should be used with caution.

The utility of SIA as a means for estimating diet composition in a generalist forager, especially species that are not omnivorous, remains equivocal (Pagani‐Núñez et al., 2017; Robinson et al., 2018). In this study, SIMM did summarize diet over a longer time period than focal observations, as models of nestling characteristics that used SIMM estimates as predictors shared significant terms with both models that used OBS_5_ and OBS_9_ estimates. SIMM estimates were also useful in identifying seasonal trends in the proportions of larvae and grasshoppers in nestling diets that were evident from observations, and supported by the literature: the larval phase of Lepidoptera is temperature‐dependent and of limited duration (Jarošík et al., 2011), and grasshoppers generally emerge later, as they have greater temperature requirements for development (Fielding, 2006). However, the high values for beetles, and low values for spiders, when estimated by SIMM may have caused inaccurate assessment of their importance to nestling growth and condition. While SIA of feathers may be a reasonable method to determine diet over longer periods of time in species such as mountain bluebirds, these estimates may have limited utility in evaluating how diet affects physical development and physiological status in individuals.

The raw data provided by provisioning recordings and SIA of nestling feathers and prey specimens were consistent, as there was clear evidence that some of the prey types we identified showed a relationship with feather δ^13^C and δ^15^N. The relative isotopic signatures of prey also corresponded to their ecology: for example, spiders, which consume insects, were the most enriched in δ^15^N, and folivorous prey, cicadas and insect larvae, the least. Consumer isotopic signatures were clearly within the bounds of mixing polygons (Figure 1), and model parameters (trophic enrichment factors) were vetted thoroughly (see Appendix 1). Theoretically, SIMM estimates produced from this process should be reliable and accurate indicators of diet composition, but in practice had limited usefulness when applied to explain metrics of growth and condition in individuals. They differed subtly but significantly from the observational data, but the small systematic differences produced resulted in some contradictory findings, such as the positive effect of date on the summary diet variable (PC1_SIMM_), which indicated that the quantity of cicadas consumed by nestlings declined, and spiders and beetles increased, over the course of the season. This is unlikely, as adult cicadas generally emerge later in the season, and have relatively high temperature requirements for activity (Jarošík et al., 2011), and beetles and spiders are a relatively poor food source that would be required in large quantities to satisfy energetic demands. If the SIMM estimates were accurate, then they would suggest that as the season progressed parents provided smaller types of prey, and avoided cicadas, which become more abundant as adults emerge in warmer temperatures. The alternative explanation, that provisioning observations are the source of these contradictory results, seems implausible given the existing knowledge regarding the foraging habits of this species (Power, 1980) and its similarity to the provisioning behaviors we documented.

While differences in diet composition between observed and SIMM estimates may seem small, they had limited utility when used to predict individual growth and condition. Additionally, the lack of clear knowledge regarding the effects of physical condition on trophic fractionation at a cellular level means that individuals in poor condition may not be reliably assessed by this method (Hobson & Clark, 1992b). This represents a substantial loss of information and statistical power. Our study shows that for SIA of dietary generalists to generate useful estimates, additional measures should be taken. SIMMs are able to find exact solutions when the number of tracers is equal to one less than the number of sources, and so better estimates are likely to result from using more tracers; while this may not be feasible for elemental tracers, recent work using fatty acids, which are much more numerous, may enable better resolution when estimating diet using mixing models in future studies.

## CONFLICT OF INTEREST

None declared.

## AUTHOR CONTRIBUTION

**Aija F. White:** Conceptualization (equal); Formal analysis (lead); Investigation (lead); Methodology (lead); Writing‐original draft (lead); Writing‐review & editing (equal). **Russell D. Dawson:** Conceptualization (equal); Formal analysis (supporting); Funding acquisition (lead); Methodology (supporting); Supervision (lead); Writing‐review & editing (equal).

## Figures and Tables

**FIGURE A1:**
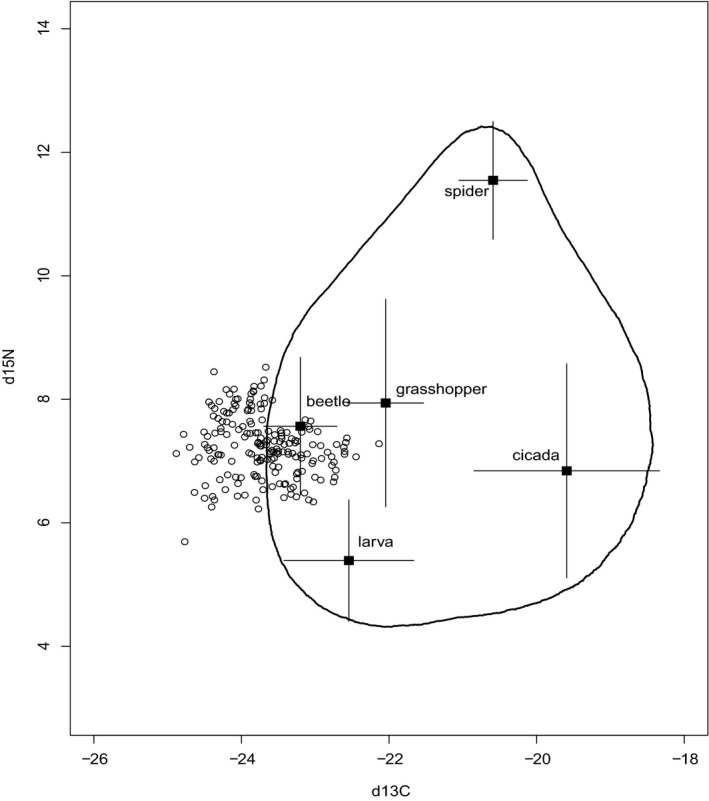
Nestling mountain bluebird (consumer) isotopic signatures (open circles), prey sources (mean ± SD; black squares), and simulated 95% mixing region (contour line), using trophic enrichment factors from Pearson et al. (2003; Δ13C = 4.3 ± 0.1 and Δ15N = 3.5 ± 0.11)

**FIGURE A2:**
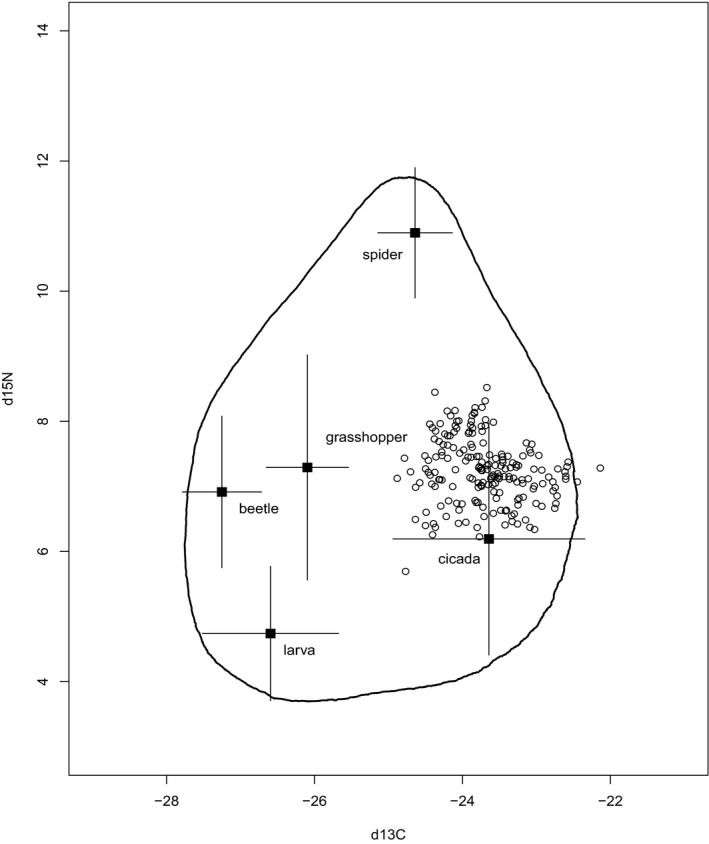
Nestling mountain bluebird (consumer) isotopic signatures (open circles), prey sources (mean ± SD; black squares), and simulated 95% mixing region (contour line), using trophic enrichment factors from Kempster et al. (2007; Δ13C = 0.25 ± 0.14 and Δ15N = 2.85 ± 0.15)

## Data Availability

The R code used for mixing models and the data used in analyses are available at Dryad: https://doi.org/10.5061/dryad.pnvx0k6nk.

## APPENDIX 1

### Macronutrient and energetic value of common prey items

We used voucher specimens and a time delay nuclear magnetic resonance body composition analyzer (Bruker MiniSpec, Billerica, MA) to determine the macronutrient profile (% fat and “lean” [protein and carbohydrate] tissue) of the five most common types of prey fed to nestling mountain bluebirds (*Sialia currucoides*), which were used to estimate their gross and metabolized energetic content (Table A1). The analyzer required a minimum of 5 g of material per sample, so we pooled as many individuals of each prey type as were needed to meet this requirement. We then calculated the mean mass per item and multiplied it by the % fat and lean to generate the masses of fat and lean tissue per item for each prey type. With these values, we estimated the gross energy content (kJ item^−1^) for each prey type using the values for kJ g^−1^ of fat (39.54) and protein (18.04) from Bell (1990). Though the analyzer we used lumps protein and carbohydrates together to estimate “lean” tissue, not protein specifically, we considered this immaterial to the caloric estimates, because proteins and carbohydrates have the same gross energy content per gram. We also generated estimates of the metabolized energy per item for each of the prey types, using metabolized energy coefficient (MEC) values drawn from the literature for insects with thick, highly sclerotized exoskeletons (beetles, cicadas, and grasshoppers; MEC = 0.70; Levey & Karasov, 1989), and for arthropods with softer, pliable cuticles (insect larvae and spiders; MEC = 0.85; Gibb, 1957).

**TABLE A1:** Mean mass per item, macronutrient composition (% fat and lean tissue), gross energy content, and estimated metabolized energy per item for each of the 5 common prey types consumed by nestling mountain bluebird (*Sialia currucoides*), from specimens collected near Williams Lake, British Columbia, Canada, in 2016. The percentages of fat and lean tissue do not sum to 100% because the remainder of each item is water mass, which has no caloric value and thus is not shown here

Prey type	Mass ($\bar{x}$ g item^−1^)	Fat (%)	Lean (%)	Gross energy (kJ item^−1^)	Metabolized energy (kJ item^−1^)
Beetle	0.15	30	28	2.54	1.78
Cicada	0.60	32	25	10.30	7.21
Grasshopper	0.46	19	30	5.95	4.16
Lepidoptera larvae	0.38	35	25	6.97	5.93
Spider	0.10	27	31	1.63	1.38

### Selection of trophic enrichment factors

In advance of constructing stable isotope mixing models, we used mixing polygon simulations to choose trophic enrichment factors (TEFs), which describe how enriched consumers are in ^13^C and ^15^N relative to their prey, using the method of Smith et al. (2013). These are Monte Carlo simulations that iteratively generate polygons from user‐provided source δ^13^C and δ^15^N values, TEFs, and their respective standard deviations. The proportion of iterations in which the isotopic signatures of each consumer fall within the 95% mixing region is determined. TEF values that result in all consumers within the mixing region in >5% of simulated polygons are considered appropriate for use in stable isotope mixing models for diet composition (Smith et al., 2013). We ran three simulations (3000 iterations each) that included all sources and consumers, with different sets of TEFs, to determine the values that best fit the study population. The three sets of TEFs we evaluated were drawn from two studies on adult passerines fed an insect‐based diet (Hobson & Bairlein, 2003; Pearson et al., 2003), and one on nestling passerines fed an omnivorous diet (Kempster et al., 2007). We chose Δ^13^C = 2.7 ± 0.1 and Δ^15^N = 4.0 ± 0.1 ( $\bar{x}\;$± SD; Figure 1; Hobson & Bairlein, 2003) as the most suitable, because all consumers fell within the mixing region in >5% of simulated polygons, unlike the results of the simulations using the other candidate TEFs (Figures A1 and A2).
